# Feasibility of a Structured Calorie-Restricted Dietary Intervention in Korean Adults with Early Type 2 Diabetes and Obesity: A Pilot Study

**DOI:** 10.3390/nu17091530

**Published:** 2025-04-30

**Authors:** Su-Jeong Park, Mee Kyung Kim, Jinyoung Kim, Ji-Yeon Choi, YoonJu Song, Hyuk-Sang Kwon

**Affiliations:** 1 Department of Food Science and Nutrition, The Catholic University of Korea, Bucheon-si 14662, Republic of Korea; yh04264@gmail.com; 2 Division of Endocrinology and Metabolism, Department of Internal Medicine, Eunpyeong St. Mary’s Hospital, College of Medicine, The Catholic University of Korea, Seoul 03312, Republic of Korea; makung@catholic.ac.kr; 3 Division of Endocrinology and Metabolism, Department of Internal Medicine, Yeouido St. Mary’s Hospital, College of Medicine, The Catholic University of Korea, Seoul 07345, Republic of Korea; j513kim.md@gmail.com; 4 Department of Nutrition, Yeouido St. Mary’s Hospital, College of Medicine, The Catholic University of Korea, Seoul 07345, Republic of Korea; topjiyoun@naver.com

**Keywords:** caloric restriction, weight loss, body weight maintenance, type 2 diabetes, obesity

## Abstract

**Background**: Calorie restriction is increasingly recognized as a strategy for glycemic management in type 2 diabetes mellitus (DM) by promoting weight loss and potentially achieving diabetes remission. This study evaluated the feasibility of a 12-week structured calorie-restricted dietary intervention based on healthy Korean dietary patterns for adults with early type 2 DM. **Methods**: Adults aged 18–60 years with a body mass index (BMI) greater than 25 kg/m^2^ and a short duration of diabetes were recruited. The intervention comprised two phases: a 6-week weight loss phase, targeting a 3% reduction every two weeks, starting with an 800 kcal/day diet and increasing by 200 kcal per phase, with all meals provided via home delivery, followed by a 6-week self-managed weight maintenance period, preceded by a one-week reintroduction phase with meals provided. **Results**: Among 27 participants, 24 completed the intervention (89% retention). Mean weight reduction at 6 weeks was 6.4 kg (7.6%), primarily achieved during Phase 1 (5.1%). By 12 weeks, the average weight reduction further improved to 7.4 kg (8.7%). Dietary adherence ranged from 80% to 90%, with energy intake closely aligning with the prescribed targets. Non-achievers exhibited a smaller initial reduction (2.5 kg vs. 3.9 kg), with the gap persisting throughout the study. Postprandial glycemic response to a mixed meal (65% carbohydrate) significantly improved from baseline to week 5. **Conclusions**: This structured dietary intervention demonstrated substantial weight reduction and improved glycemic response in adults with type 2 DM, highlighting its feasibility and effectiveness as a guided strategy for weight management.

## 1. Introduction

With the number of people with type 2 diabetes mellitus (DM) rising worldwide, it has become a major public health issue in South Korea. The prevalence of diabetes among Koreans aged 30 or more significantly increased from 9.1% in 2005 to 15.5% in 2021–2022, according to national survey data [1,2]. There is a strong link between obesity and type 2 diabetes in Korea. Obesity prevalence has continuously increased over the past decade, with 55.1% of adults with diabetes also being obese [2,3]. As the population’s lifestyle has shifted towards less physical activity and a more Westernized diet, obesity rates have increased, subsequently leading to a higher prevalence of type 2 diabetes [4,5].

Several strategies are currently used for glycemic management in type 2 DM, with the initial approach focusing on lifestyle changes, including physical activity and dietary recommendations. The most recommended nutritional guidelines for diabetes involve restricting dietary carbohydrates and increasing dietary fiber, with a greater focus on the quality of dietary fat and carbohydrates rather than the quantity of these macronutrients [6,7]. Low-carbohydrate diets have been effective in glycemic management. In a meta-analysis of 23 clinical trials for at least 12 weeks among adults with type 2 diabetes, low-carbohydrate diets demonstrated efficacy and safety for type 2 diabetes remission, showing significant remission of diabetes without adverse consequences compared with other diets, including low-fat diets [8]. However, dietary modifications are not always successful, especially in the case of severe obesity.

Calorie restriction is recommended to promote weight loss for adults with type 2 diabetes and has been shown to improve insulin sensitivity, reduce lipotoxicity and inflammation, and enhance beta-cell function [9,10,11]. These mechanisms collectively contribute to better glucose control and can potentially help adults with type 2 diabetes achieve normal glucose levels [12,13].

Among these, very low-calorie diets (VLCD), typically defined as providing less than 800 calories per day, have been shown to improve glycemic response [14]. A systematic review of VLCD in adults with type 2 diabetes showed significant reductions in both glycated hemoglobin A1c (HbA1c) and fasting glucose following the VLCD [15]. Additionally, a VLCD for 3 months, followed by food reintroduction and then structured support, showed nearly half of people with type 2 diabetes achieving remission to a non-diabetic state [16] and 61% achieving normoglycemia at 12 months [17]. Meta-analyses of 15 studies showed that the combination of very low-calorie and ketogenic diets (about 500–800 kcal/day with less than 50 g of carbohydrate) resulted in significant reductions in fasting glucose, HbA1c, and HOMA-IR from baseline after short- or long-term weight loss intervention, and this approach has been adopted in European Guidelines for obesity management [18].

Although there is increasing interest in the use of VLCD or their combination with ketogenic diets, few studies have been conducted in Asian adults. Traditionally, the Asian population consumes high-carbohydrate diets. In particular, Korean adults follow a rice-based diet with a variety of plant foods [19], resulting in carbohydrate intake reported to be 62.4% in adults and even higher at 66.3% in adults with diabetes [2]. The aim of this pilot study was to assess the feasibility of a 12-week structured calorie-restricted dietary intervention in Korean adults with early type 2 diabetes. The program began with a VCLD (800 kcal/day) and progressively increased in energy intake across phases, ultimately transitioning to a healthy diet characterized by moderate carbohydrate intake and a variety of foods based on the Korean dietary patterns.

## 2. Materials and Methods

### 2.1. Study Design and Participants

The study was designed as a 12-week, open-label, controlled trial as a single-center study. The 12-week intervention consisted of an integrated structured weight management intervention, with a 6-week weight loss period starting with a VLCD and full food provision, followed by a 6-week maintenance period during which participants independently managed their usual diets. This study is part of the CREDO-K study (Caloric Restriction on Diabetes Remission of Korean Adults with Obesity) [20].

Participants were recruited from The Catholic University of Korea, Yeouido ST. Mary’s Hospital Endocrinology. Eligible participants were aged 18–60 years, had been diagnosed with type 2 DM within the previous 6 years, and had a body mass index (BMI) greater than 25 kg/m^2^. Exclusion criteria included current insulin use, HbA1c ≥ 75 mmol/mol (9%), >5 kg weight loss within the previous 6 months, current treatment with anti-obesity drugs, pregnant women, individuals with heart failure, liver failure, renal failure, chronic obstructive disease, known malignancy history, cardiovascular disease (myocardial infarction or stroke) within the previous 6 months, acute infection or treatment within the past 3 months requiring hospitalization, endocrine disease affecting blood glycemic response within 6 months, or specific food allergies.

All participants signed informed consent prior to any study-related procedures. The study protocol was approved by the institutional review board of The Catholic University of Korea, Yeouido ST. Mary’s Hospital (SC23ENSE0036), and registered on ClinicalTrials.gov (NCT05754775).

### 2.2. Dietary Protocol

**Figure 1:**
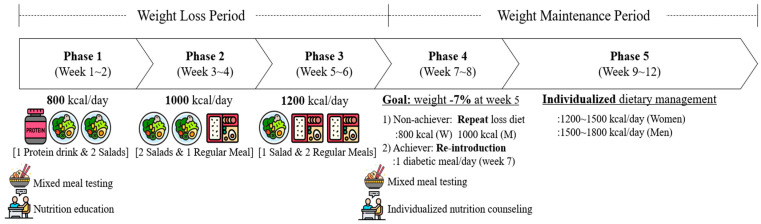
Overview of the calorie restriction intervention in the study. The program consisted of a 6-week weight loss period followed by a 6-week weight maintenance period. Meals were provided during the weight loss period, starting at 800 kcal/day and increasing by 200 kcal with each phase, including the transition (Phase 4). During the maintenance period, all participants managed their own diets. Mixed meal testing was conducted at weeks 0 and 5, nutrition education was provided at week 0, and individualized nutrition counseling was offered at week 5.

The dietary goal of this intervention was to promote a healthy diet with moderate carbohydrate intake and a variety of foods based on the Korean dietary pattern. The nutritional target aimed to reduce daily intake to 40% to 60% of total energy from carbohydrates, with less than 10% of energy from total sugar intake. The daily energy intake is set at 800~1200 kcal during the weight loss period, depending on the phase, and was individually recommended at 1200~1500 kcal for women and 1500~1800 kcal during the weight maintenance period. Two nutritional education sessions were conducted. At baseline, the first session provided information about calorie restriction diets and the overall dietary regimen, based on typical nutrition education for type 2 diabetes by registered dietitians. The second session took place individually at the beginning of the weight maintenance period to help participants build their own healthy diets based on their 6 weeks of experience, promoting the maintenance of reduced weight by the registered dietitian. A mixed meal, consisting of a typical Korean meal with rice and side dishes, was provided at both the pre-intervention week (Week 0) and the end of the weight loss period (Week 5) to evaluate improvements in postprandial glycemic response (Figure 1).

Throughout the weight loss period, participants were provided with three daily meals free of charge to fully cover daily energy needs. All food and beverages were delivered to each participant individually three times per week through GREATING service (HYUNDAI GREEN FOOD Co., Yongin, Korea). All menus were composed of varied food ingredients, except for the protein drink. A sample weekly menu is presented in Table A1.

#### 2.2.1. Weight Loss Period

The weight loss period was divided into 3 phases of calorie restriction, each lasting 2 weeks, with the goal of achieving a 3% reduction in body weight from baseline. Phase 1 targeted 800 kcal per day and typically consisted of one meal-replacement protein drink (160 kcal; 18 g carbohydrate, 27 g protein) and two salads (48% carbohydrate, 23% protein, 29% fat). Phase 2 targeted 1000 kcal per day and typically comprised two salads (or one salad and one light meal) and one regular meal (50% carbohydrate, 18% protein, and 32% fat). Phase 3 targeted 1200 kcal per day and typically consisted of one salad and two regular meals (51% carbohydrate, 16% protein, and 33% fat). During this period, participants were allowed to consume unsweetened tea or coffee and non-starchy vegetables such as cucumber, broccoli, and cabbage if they felt hungry.

#### 2.2.2. Weight Maintenance Period

The weight maintenance period was divided into 2 phases, with the goal of reintroducing a healthy diet that would maintain the reduced weight while providing recommended energy intake with the nutritional targets established during the weight loss period. In Phase 4, a decision was made whether to continue the weight loss diet or move to the reintroduction phase. The cutoff was a 7% weight reduction by week 5 for food delivery. Participants who did not achieve this goal were provided with the weight-loss diet again, targeting 800 kcal for women and 1000 kcal for men during phase 4. Participants who reached the goal received only one meal per day during the first week of Phase 4 and were then allowed to plan their own meals freely. Phase 5 targeted 1200~1500 kcal for women and 1500~1800 kcal for men, consistent with clinical guidelines for weight loss [21]. Based on their 6-week experience, they were encouraged to build their own meals without provided food to support long-term maintenance of a healthy diet.

### 2.3. Outcome Measures

#### 2.3.1. Anthropometric Measures and Body Composition

All participants were provided with a weight scale at baseline and asked to report their daily weight via mobile app. Participants’ height was measured at baseline, and body composition was measured using bioelectrical impedance analysis (InBody Co., Seoul, Republic of Korea) at the baseline, week 6, and week 12, for a total of three measurements. BMI was calculated as weight (kg) divided by height squared (m^2^), using the baseline measurements.

The goal for weight reduction was set at 3% per phase, aiming for a total of 9% weight reduction by the end of the weight loss period. “Achievers” were defined as those who met the weight reduction goal of 7% by week 5 due to the food delivery schedule. “Non-achievers” were those who did not meet this weight reduction goal.

#### 2.3.2. Nutrient Intake and Dietary Adherence

During the 12-week intervention, all participants were required to report their daily dietary records via a mobile app. Each morning, participants logged in the previous day’s meals, including provided meals and any extra foods and beverages consumed during the weight loss periods. For the weight maintenance periods, the dietary records included the type, amount, and time of all foods and beverages consumed. If dietary reports lacked information, the dietitian requested further details. This app also served as a tracking tool to help participants adhere to the dietary regimen. All dietary records were inputted, and nutrient intake was calculated using the Computer Aided Nutritional Analysis program, a web-based nutrient assessment software developed by the Korean Nutrition Society.

Binary categorical measures were created to evaluate dietary adherence during the study period. “Successful” was defined as consuming daily energy intake within 10% of the given value. “Not successful” was defined as consuming daily energy intake more than 10% above the given value in each phase. For the weight maintenance, exceeding 10% of 1500 kcal per day for women and 1800 kcal per day for men was used as the cutoff.

#### 2.3.3. Postprandial Glycemic Response of Mixed Meal

A standardized mixed meal was designed to evaluate the response to a typical Korean meal. The meal consisted of a mixed rice bowl, known as bibimbap, with a nutrient content of 582 kcal (2436 kJ), providing 65% of energy from carbohydrate, 10.5% from protein, and 24.0% from fat. Postprandial glucose levels were measured for up to 120 min using a continuous glucose monitoring system (FreeStyle Libre, Abbott Korea., Seoul, Korea). Mixed meal testing was conducted twice before the intervention and after 5 weeks of calorie restriction. The individual glycemic response to the standardized meals was used as reference data for individualized nutritional counseling. The area under the curve (AUC) for glucose responses over 120 min was calculated using the trapezoidal method.

#### 2.3.4. Other Measures

The basic and clinical information was obtained at baseline. The clinical information included diabetes profile, blood measures, and comorbidities. The diabetes profile included the duration of diabetes, fasting blood glucose levels, and HbA1c at baseline. Blood measures included serum total cholesterol, LDL-cholesterol, HDL-cholesterol and triglycerides. Comorbidities and medication at baseline were also included.

### 2.4. Statistical Analysis

Continuous variables are presented as mean ± standard deviation, and categorical variables are presented as (*n*, (%)). Differences in the basic characteristics of participants according to sex were examined by Wilcoxon nonparametric tests. Within-group evaluations of changes in outcome variables were performed with paired *t*-tests for parametric variables or paired Wilcoxon signed-rank tests for nonparametric variables. Between-group comparisons of changes in outcomes were performed with independent *t*-tests for parametric variables or Wilcoxon rank-sum tests for nonparametric variables. All statistical analyses were performed using SAS 9.4 (SAS Institute Inc., Cary, NC, USA), and statistical significance was considered when *p* < 0.05.

## 3. Results

### 3.1. Participants

Study participants were enrolled weekly in groups of 3 or 4 participants, with each group following its own dietary schedule. Two independent cycles were conducted. Participants in the first cycle were recruited between July 2023 and September 2023, with the cycle concluding in November 2023. The second cycle, which followed the same format as the first, took place from December 2024 and ended in March 2024. Among twenty-seven participants who started the study at baseline, three dropped out due to schedule conflicts and low compliance. Thus, a final total of twenty-four participants completed the 12-week dietary intervention.

Baseline characteristics of study participants are described in Table 1. The average age of participants was 42.2 years, with 50% being male. The average BMI was 29.6 kg/m^2^, with a significant difference between men and women (31.5 kg/m^2^ in men vs. 27.8 kg/m^2^ in women). The average duration of diabetes was 2.5 years. At baseline, the average fasting blood glucose level was 127.8 mg/dL, and the average hemoglobin A1c level was 53.1 mmol/mol (7.0%). There were no significant differences between men and women in these measures. The average total cholesterol level was 150 mg/dL, HDL-cholesterol was 53.0 mg/dL, and triglycerides were 138.5 mg/dL. Additionally, there was no significant difference in any clinical measures between men and women. The prevalence of hypertension was 66.7% in men and 25.0% in women; however, there was no significant difference in the prevalence of dyslipidemia.

### 3.2. Nutrient Intake and Dietary Adherence

The average nutrient intake during the study period is presented in Table 2. The average energy intake was 828.0 kcal (3465.8 kJ) in Phase 1, 964.5 kcal (4037.5 kJ) in Phase 2, and 1172.3 kcal (4907.1 kJ) in Phase 3, which is close to the targeted calorie intake of 800 kcal in Phase 1, 1000 kcal in Phase 2, and 1200 kcal in Phase 3. During the maintenance period, Phase 4 served as a transitional phase in which 14 participants who achieved their weight reduction goals within the first 6 weeks moved to the reintroduction phase, where one meal was provided. In contrast, 10 participants, classified as non-achievers, repeated the calorie-restriction diet (800 kcal for women and 1000 kcal for men) during Phase 4. The average energy intake during this phase was 1140.8 kcal (4775.6 kJ), with the repeated group consuming an average of 851.9 kcal (3566.2 kJ) and the reintroduction group consuming 1347.2 kcal (5639.4 kJ). In Phase 5, participants resumed their own diet, and the average nutrient intake was 1287.3 kcal (5388.7 kJ), with macronutrient proportions of carbohydrate: protein: fat = 45.2: 21.3: 33.5%. Sugar intake was 6.4% and 5.5% of energy and saturated fat was 8.5% and 8.7% of energy in each group, both of which are less than 10%. The macronutrient composition during the maintenance period was similar to that of the weight loss period.

**Figure A1:**
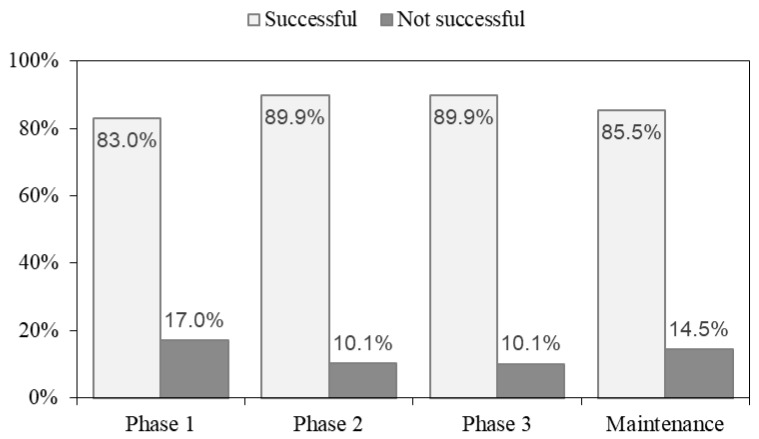
Dietary adherence of participants to daily energy intake during the study period. ‘Successful’ participants consumed energy intake within 10% of the target, while others were classified as ‘Not successful’.

The dietary adherence during the study period is presented in Figure A1. In the weight loss period, during which all meals were provided, the average rate of dietary adherence was 83.0% in Phase 1, 89.9% in Phase 2, and 89.9% in Phase 3. In the maintenance period, during which participants individually pursued healthy diets, the average rate of dietary adherence among achievers was 85.5%.

### 3.3. Changes in Body Weight

**Figure 2:**
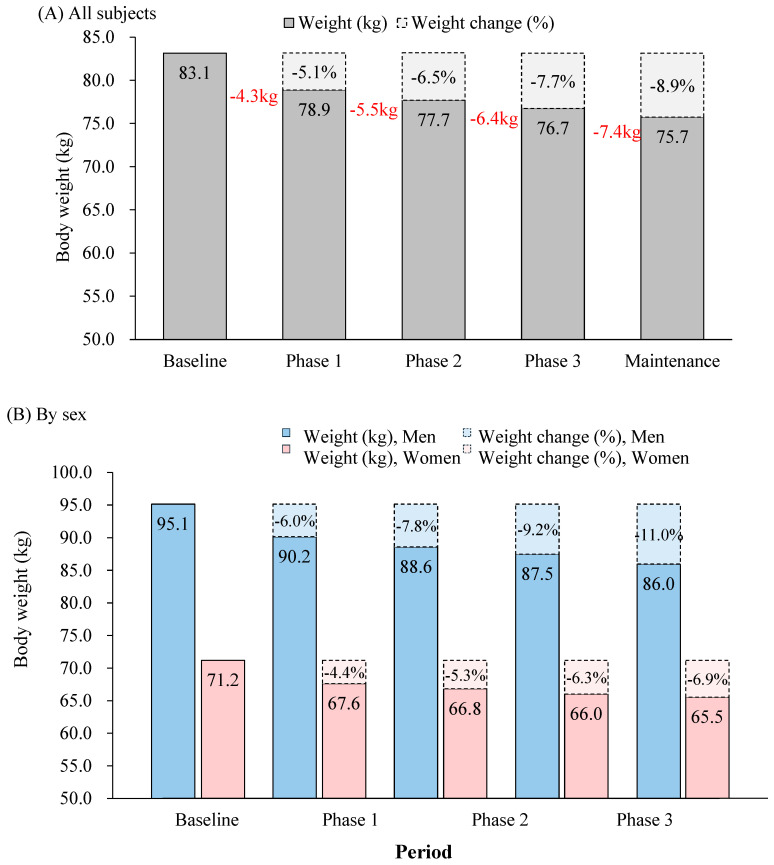
Changes in body weight and percentage from the baseline by phase during the weight loss period and the average during the maintenance periods of all subjects (**A**) and by sex (**B**).

The impact of the calorie-restricted dietary regimen on body weight during the entire study period is presented in Figure 2. The average body weight was 83.1 kg at baseline and gradually reduced to 78.9 kg in Phase 1, 77.7 kg in Phase 2, 76.7 kg in Phase 3, and 75.7 kg during the maintenance period. The actual weight reduction was 4.3 kg (5.1%) in Phase 1, which was the largest decrease. The extent of weight loss gradually became smaller over the subsequent phases. The total weight reduction at the end of the weight loss period was 6.4 kg (7.6%), and by the end of the weight maintenance period, it was 7.4 kg (8.7%).

### 3.4. Weight Change and Nutrient Intake by Achievement of Weight Reduction Goals

**Figure 3:**
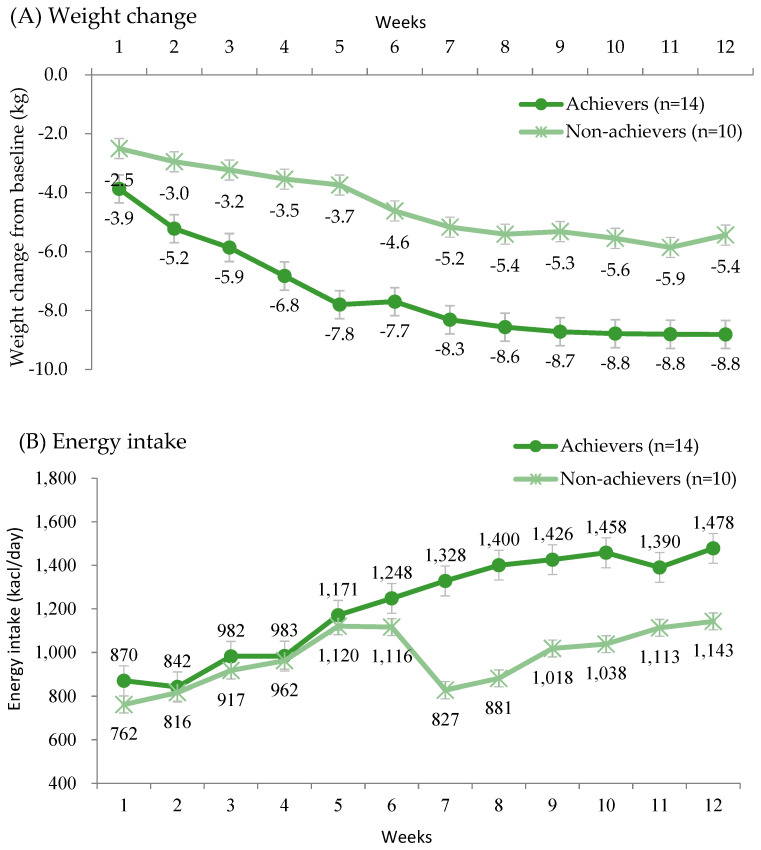
Changes in body weight from baseline during the study period. (**A**) Weekly weight changes by participants based on achievement of the weight reduction goal. (**B**) Average daily energy intake consumed by participants based on achievement of the weight reduction goal. Achievers were defined as participants who met the 7% weight reduction goal by week 5. Those who did not achieve this goal (‘Non-achievers’) were provided with the weight-loss diet again—800 kcal for women and 1000 kcal for men during phase 4 (weeks 7~8) to support continued weight reduction. Mean values of weekly weight change and daily energy intake (except for week 5) were significantly different between groups by the Wilcoxon rank-sum test (*p* < 0.05).

The weight change and energy intake by group based on weight reduction goal achievement are presented in Figure 3. From the first week, weight changes between groups began to diverge, with a 3.9 kg reduction in the achiever group compared to a 2.5 kg reduction in the non-achiever group. This difference persisted throughout the study. By the end of the study, the achiever group had reduced their body weight by 8.8 kg, while the non-achiever group had reduced their weight by 5.4 kg. During the weight loss period, the non-achiever group reported a lower average energy intake compared to the achiever group. At week 7, marking the beginning of the weight maintenance period, the non-achiever group continued the repeated calorie-restriction diets, consuming an average of 827 kcal (3566 kJ), compared to 1328 kcal (5639 kJ) in the achiever group. For the achiever group, the energy intake closely aligned with the targeted intake.

### 3.5. Postprandial Glycemic Response

**Figure 4:**
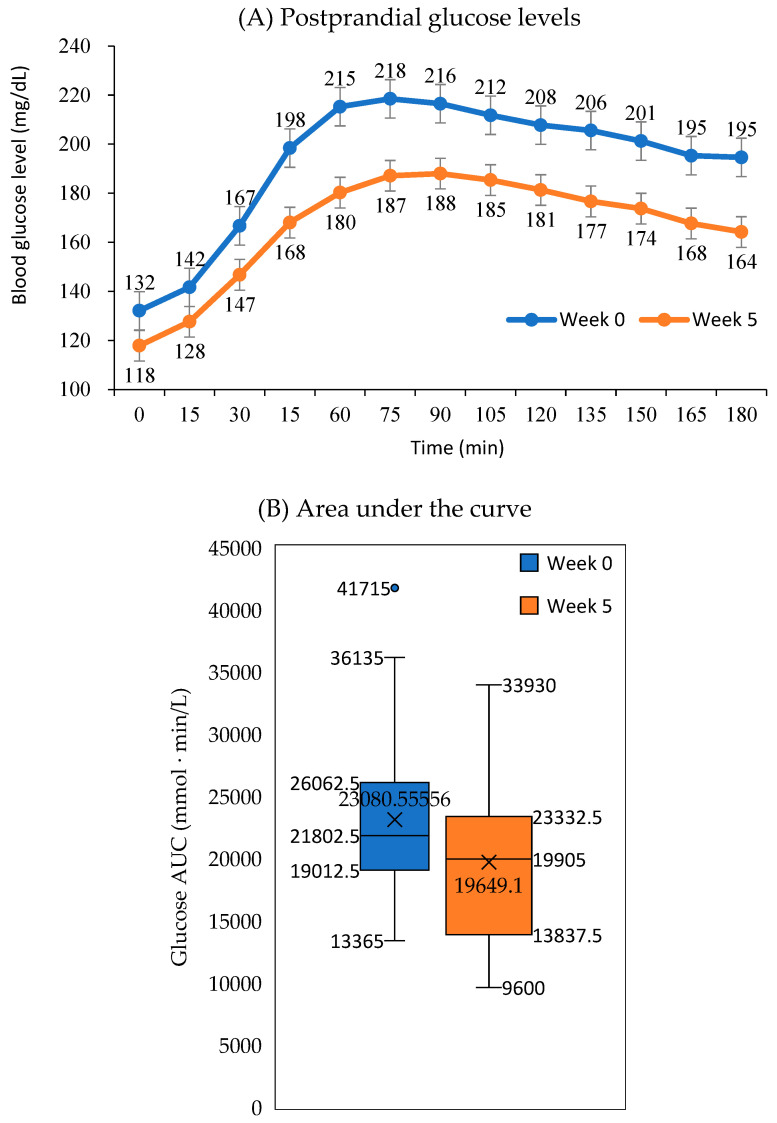
Changes in postprandial glycemic response to a mixed meal with 65% of energy from carbohydrate between week 0 and week 5 using continuous glucose monitoring system. (**A**) Postprandial glucose levels after meal. (**B**) Glucose area under the curve over (AUC) 120 min. Mixed meal testing was performed at both times to compare glycemic response before and after the very low-calorie diet. The postprandial glucose levels at each time point (except for 0 and 15 min), as well as the AUC values over 120 min, were significantly different between week 0 and week 5 within participants by Wilcoxon signed-rank test (*p* < 0.05).

Postprandial glycemic responses to a mixed meal in week 0 and week 5 are presented in Figure 4. The fasting glucose level was 132 mg/dL in week 0, which decreased to 118 mg/dL by week 5. The peak value, observed 75 min after the meal, was 218 mg/dL in week 0 but dropped to 187 mg/dL in week 5. Additionally, the glucose levels at 180 min after a meal decreased from 195 mg/dL in week 0 to 164 mg/dL in week 5. Overall, the postprandial glycemic response improved after 5 weeks of calorie restriction. The average AUC was 1281 mmol∙ min/L at week 0, significantly decreasing to 1091 mmol∙ min/L by week 5.

## 4. Discussion

In this study of a structured calorie-restricted dietary intervention, participants achieved significant weight loss and improved glycemic response. This is the first study to examine the feasibility of a VLCD based on a structured healthy dietary pattern, rather than total meal replacements, in Korean adults with type 2 diabetes. The findings demonstrate that a 6-week calorie restriction with a well-designed menu can be effectively implemented and provide a foundation for self-managed dietary maintenance.

We found that the greatest reduction in weight occurred during Phase 1 with an 800 kcal diet. It is well known that VLCD (800 kcal/day) leads to greater short-term weight loss compared to other diets [14,22]. A study of 6-month calorie restriction revealed that the VLCD (890 kcal) group showed a 13.9% weight reduction, which is significantly greater than that in the control group (1.0%) and even greater than that in the low-calorie diet group with a 25% calorie restriction of baseline energy requirements (10.4%) [23].

While VLCD can lead to considerable short-term weight loss, maintaining that loss remains a challenge. For successful maintenance, it is crucial to recognize that weight loss and weight-loss maintenance are two distinct physiological states [24]. Weight loss induces metabolic adaptations that result in changes such as reduced energy expenditure and can also be influenced by the surrounding environment. The DiRECT study, a primary care-led weight management program for adults with type 2 diabetes, comprised a VLCD for 3–5 months, followed by a food reintroduction period for 2–8 weeks and structured support for long-term weight maintenance [16]. The study reported a 10.0 kg weight reduction, with nearly half of participants in remission to a non-diabetic state and off antidiabetic drugs one year later [16], with 36% still in remission at two years [25]. The DIADEM-I trial, using the same format with a VLCD for 12 weeks, followed by gradual food reintroduction combined with physical activity support in a weight-loss maintenance phase, reported that a mean reduction in weight of 11.98 kg at 12 months [17]. This indicates that a VLCD can be used as an initial protocol in a structured weight loss program, which is effective for maintaining reduced weight in the long term.

Unlike these studies, our initial VLCD period lasted 2 weeks, followed by an addition of 200 kcals every 2 weeks. According to a study of a VLCD until achievement of 10% weight loss, the average time was 11.9 weeks [26], similar to the DiRECT [16] and DIADEM-I trials [17]. We observed the largest reduction in Phase 1 but continued reduction in the following phase, resulting in a total weight loss of 6.4 kg (7.6%) from the baseline and 6 weeks. This weight loss was maintained during the self-selected dietary management period, resulting in a total weight loss of 7.4 kg (8.7%) at 12 weeks.

Additionally, VLCD and total meal replacements have been reported as the most effective approach for diabetes remission [27]. However, this study did not provide total meal replacement during the VLCD phase. While a protein drink was included, the other two meals consisted of salad or a light dish. We intentionally designed a menu with a variety of ingredients, and all meals were delivered regularly to participants as fresh as possible, either frozen or refrigerated. This approach not only relieved participants from meal preparation but also allowed them to experience a variety of dishes, which can be beneficial for the maintenance phase.

This was made possible by the availability of many products for therapeutic meals in Korea. In 2022, the Korea Ministry of Food and Drug Safety established standards for therapeutic meals, an additional category distinct from formula foods, to support the prevention and management of chronic disease at home. For diabetes, a therapeutic meal for diabetes should contain 500~800 kcal, less than 10% of energy from sugar, over 18 g of protein, less than 10% of energy from saturated fat, and a balanced mix of grain, fish, meat, and vegetables. Home-delivered meals for diabetes have shown a glucose-lowering effect in Korea, with significant reductions in blood glucose levels and HbA1c when two out of three main meals were replaced with these therapeutic meals [28].

In the maintenance period, participants managed their own diets. At the beginning of this period, individual nutrition education was provided, focusing on meal planning rather than selecting specific dietary strategies. A high-protein diet was found to be more beneficial for maintaining reduced weight over 12 months following an average weight loss of 11.2 kg through an 8-week VLCD (800–880 kcal) compared to low-glycemic diets [29]. However, there is no single best diet strategy for weight maintenance [30], so individualized approaches are recommended. Having experienced 6 weeks of a healthy diet menu, participants knew what to choose, but meal planning for social gatherings remained challenging. Indeed, during the maintenance period, the average energy intake was 1287.3 kcal (5388.7 kJ), with carbohydrate intake at 45.2%. This is relatively low compared to the average intake of Korean adults (62.4%) and similar to low-carbohydrate diets in Korean adults [31].

We evaluated dietary adherence based on the calories consumed by phase. Adherence, defined as being within 10% of the targeted energy intake, was 83% in phase 1 and 90% in Phases 2 and 3, which is quite favorable. Participants were also satisfied with all the meals provided. The gradual weight reduction during this period demonstrated the feasibility of the dietary protocol. Interestingly, the non-achiever group reported a lower average energy intake than the achiever group during the weight loss period, and their intake remained significantly lower during the weight maintenance period following repeated low-calorie diets in the transitional phase 4. Given that energy intake was self-reported, this discrepancy may be influenced by factors such as underreporting. Previous studies have shown that participants with obesity tend to underreport energy intake by 37%, with 26% attributed to undereating and 12% to under-recording [32]. Additionally, underreporting has been linked to body image perception and dissatisfaction [33].

Despite the non-achiever groups showing a smaller reduction in weight change and lower reported energy intake, they still achieved modest weight loss. However, the trend in weight change over the study period indicated that the non-achiever group had a smaller initial weight reduction (2.5 kg vs. 3.9 kg), and the gap in weight change between groups persisted throughout the study. This suggests that successful weight reduction is heavily dependent on proper adherence and implementation during the initial phase, even though the first 800 kcal diet phase is often the most challenging for participants.

Regardless of weight goal achievement, the average weight reduction was 7.4 kg (8.7%), supporting the overall feasibility of this intervention. In addition, we previously reported diabetes remission outcomes in this population, with 75% (18 out of 24) of participants achieving remission, defined as HbA1c < 6.5%, through weight reduction [20].

No serious adverse events were reported during the intervention. There was no event of hypoglycemia throughout the study period. One patient developed a coronavirus infection, which resolved with conservative treatment. The most common adverse events were hunger and frailty, primarily occurring during the first phase of the VLCD.

Our study has several limitations. One limitation is the small sample size, which may impact the generalizability of the results. Despite this, it is notable that this is the first study in Korea to conduct a weight-loss intervention via calorie restriction in adults with type 2 diabetes in Korea. Another limitation is the lack of objective measurement of physical activity, which could influence weight changes. Although participants were instructed not to alter their usual lifestyles or increase physical activity during the study period, activity levels were not objectively tracked. The reliance on self-report may have missed unintentional or unreported increases in physical activity, particularly among participants in the Achiever group. Additionally, dietary adherence was based on self-reporting, which is inherently prone to underreporting and may have led to underreporting of additional food consumption. These limitations may partially explain the greater weight loss observed in the Achiever group, despite their higher reported energy intake. Nonetheless, all participants achieved weight reduction, although the extent of weight loss varied.

## 5. Conclusions

In summary, this pilot feasibility trial of a structured calorie-restricted dietary intervention demonstrated significant weight reduction and improved glycemic response in adults with early type 2 diabetes. The dietary regimen of calorie restriction, which began with an 800 kcal/day intake and increased by 200 kcal every two weeks, was both effective and well-tolerated. The structured dietary intervention—comprising a caloric restriction phase with a variety of healthy; provided meals; followed by a self-managed reintroduction phase—empowered participants to develop dietary self-management skills. These findings confirm the feasibility and potential of this approach as a guided strategy for long-term weight management.

## Figures and Tables

**Table 1:** Basic characteristics of study participants.

	Total (*n* = 24)	Men (*n* = 12)	Women (*n* = 12)	*p*-Value ^1^
Age (years)	42.2 ± 10.9	43.3 ± 10.2	41.1 ± 12.1	0.7069
Body weight (kg)	83.1 ± 15	95.1 ± 10	71.2 ± 7.6	<0.0001
BMI (kg/m^2^)	29.6 ± 3.9	31.5 ± 4.3	27.8 ± 2.6	0.0464
Waist circumference (cm)	97.3 ± 11.3	105.6 ± 9	89.0 ± 6.0	<0.0001
Muscle mass (kg)	29.7 ± 6.8	35.5 ± 3.5	23.9 ± 3.4	<0.0001
Fat mass (kg)	29.7 ± 7.9	32 ± 9.6	27.3 ± 5	0.2252
Body fat percent (%)	38.6 ± 13.9	39 ± 19.4	38.3 ± 5.1	0.2366
Clinical measures				
Duration of diabetes (years)	2.5 ± 2.0	1.8 ± 1.3	3.2 ± 2.3	0.1749
Fasting blood glucose (mg/dL)	127.8 ± 31.9	123.8 ± 38.6	131.8 ± 24.5	0.1407
HbA1c (mmol/mol)	53.1 ± 13.5	51.2 ± 14.5	54.9 ± 12.6	0.3545
(HbA1c, %)	(7.0 ± 1.2)	(6.8 ± 1.3)	(7.2 ± 1.2)	
Total cholesterol (mg/dL)	150 ± 24.9	143.7 ± 24.6	156.3 ± 24.6	0.2251
Low-density lipoprotein cholesterol (mg/dL)	80 ± 19.8	75.3 ± 22.8	84.7 ± 15.8	0.2034
High-density lipoprotein cholesterol (mg/dL)	53 ± 10.1	51.4 ± 11	54.5 ± 9.4	0.3708
Triglycerides (mg/dL)	138.5 ± 65.4	134.4 ± 65.5	142.6 ± 68	0.8625
Systolic blood pressure (mmHg)	130.2 ± 15.4	132 ± 17.4	128.3 ± 13.6	0.7503
Diastolic blood pressure (mmHg)	82.4 ± 11.5	84.2 ± 11.1	80.7 ± 12	0.4184
Comorbidities				
Hypertension (n, %)	11 (45.8)	8 (66.7)	3 (25.0)	0.0486
Dyslipidemia (n, %)	23 (95.8)	12 (100.0)	11 (91.7)	0.3593

^1^ *p*-value by Wilcoxon test.

**Table 2:** The average nutrient intake during weight loss and maintenance periods.

Nutrients	Weight Loss Period			Weight Maintenance Period			
	Phase 1	Phase 2	Phase 3	Phase 4		Phase 5	
				Achiever (*n* = 14)	Non-Achiever (*n* = 10)	Achiever (*n* = 14)	Non-Achiever (*n* = 10)
Energy (kcal/day) (Mean ± SD)	828.0 ± 124.4	964.5 ± 84.7	1172.3 ± 133.7	1347.2 ± 266.8	851.9 ± 130.4	1436.9 ± 305.1	1077.9 ± 209.3
Carbohydrate (g)	104.3 ± 13.3	125.6 ± 17.9	154.4 ± 19.6	153.9 ± 39	115 ± 15.9	160.7 ± 36.7	113 ± 37.1
Sugar (g)	19.1 ± 3.5	20.6 ± 5.1	22.7 ± 6.5	25.2 ± 14.5	18.7 ± 2.9	22.9 ± 8.2	14.6 ± 7.7
Protein (g)	48.8 ± 9.9	46.8 ± 7.4	48.8 ± 8.6	70.4 ± 13	42 ± 7.9	73.8 ± 13.5	54.7 ± 11.8
Fat (g)	29 ± 5.8	35.7 ± 3.9	44.5 ± 6.4	49.8 ± 13.1	28.8 ± 6	53 ± 13.3	43.5 ± 12.9
Saturated fat (g)	5.5 ± 2.0	6.6 ± 1.2	8.5 ± 2.1	11.7 ± 3	5.8 ± 1.6	13.5 ± 3.5	11.2 ± 5.1
Percent of energy							
Carbohydrate (%)	47.9 ± 3.5	49.5 ± 4.3	51 ± 3.2	46.4 ± 6.2	52.4 ± 2.9	46 ± 5.1	44.1 ± 11.4
Sugar (%)	8.8 ± 1.4	8.1 ± 1.5	7.4 ± 1.4	7.1 ± 2.8	8.6 ± 1.5	6.4 ± 1.8	5.5 ± 2.5
Protein (%)	22.2 ± 2.6	18.6 ± 3.1	16 ± 1.6	21.4 ± 4.4	18.7 ± 1.7	21.5 ± 2.4	21 ± 4.5
Fat (%)	29.9 ± 4.1	31.8 ± 2.6	33 ± 2.8	32.2 ± 4.2	28.9 ± 2.6	32.5 ± 3.7	34.9 ± 7.4
Saturated fat (%)	5.6 ± 1.5	5.9 ± 1.2	6.3 ± 1.3	7.5 ± 1.2	5.7 ± 0.8	8.2 ± 0.9	8.7 ± 2.2

## Data Availability

The datasets generated and analyzed during the current study are not publicly available due to institutional policies on participant confidentiality but are available from the corresponding author upon reasonable request.

## Appendix A

**Table A1:** The sample menu by phase in the weight loss period.

	800 kcal	1000 kcal	1200 kcal
Day 1	Protein Drink	Chicken Breast Salad	Vietnamese Spring Rolls Salad
	Fruit Cashew nut Salad	Mapa eggplant with Palbo rice	Hot Chicken Bulgogi and Brown Chickpeas Rice
	Stir-fried pork and Sweet potato barley rice	Champignon Gravy Chicken Set	Soft Tofu Gang-doenjang and Green tea Seagrass Rice
Day 2	Protein Drink	Protein Drink	Grilled Potato Cheese Salad
	Yangjanpi Salad	Taco Salad	Ssamjang Fried Pork and Brown Barley Rice
	Mapa eggplant with Palbo rice	Sichuan Steamed Zucchini Set	Yangjanpi Salad
Day 3	Protein Drink	Poke Bowls Salad	Taco Salad
	Basil Webfood Octopus Salad	Bacon Kimchi Fried Rice	Stir-fried Squid and Brown Oat Rice
	Gyudong with Brown Oat rice	Sichuan Eggplant Rice Set	Egg and Bulgogi Salad
Day 4	Protein Drink	Protein Drink	Poke Bowls Salad
	Bun Cha Salad	Chicken Steak Salad	Mediterranean Rice Set
	Walnut Bibimbap	Greek Braised Pork Set	Bulgogi and Korean Vegetables and Brown Barley Rice
Day 5	Protein Drink	Protein Drink	Root Vegetables Chicken Salad
	Tofu Salad	Yuringi Salad	Bulgogi and Korean Vegetables and Brown Barley Rice
	Bacon kimchi fried rice	Japanese Hamburg Curry Set	Sriracha sauce Chicken Salad
Day 6	Protein Drink	Short-fruit Pimpinella Meat Pasta Salad	Conchiglie Jambon Salad
	Potato Blueberry Compote Salad	Walnut Bibimbap	Vegan Hamburg Steak and Grilled Emergency crop
	Pumpkin Bean cream Risotto and Fried chicken	Jambon Tortilla Salad	Grilled Chicken and Brown Chickpeas Rice
Day 7	Protein Drink	Protein Drink	Blueberry Compote Salad
	Ricotta Granola Salad	Blueberry Compote Salad	Abalone Rice and Beef bone Dried Pollak Soup
	Chicken Cashew nut Salad and Brown Oat rice	Chickpeas Cream Chicken Steak and Taco Pilaf	Chicken Casew nut Curry and Brown Oat Rice

**Table A2:** The average nutrient intake during weight maintenance periods by sex.

Nutrients	Phase 4				Phase 5			
	Men (*n* = 12)		Women (*n* = 12)		Men (*n* = 12)		Women (*n* = 12)	
	Achiever (*n* = 8)	Non-Achiever (*n* = 4)	Achiever (*n* = 6)	Non-Achiever (*n* = 6)	Achiever (*n* = 8)	Non-Achiever (*n* = 4)	Achiever (*n* = 6)	Non-Achiever (*n* = 6)
Energy (kcal/day)	1345 ± 246.4	983.8 ± 68	1350.2 ± 316.4	764 ± 68	1524.9 ± 308.7	1169.9 ± 186.2	1319.6 ± 282.4	1016.5 ± 216.1
Carbohydrate (g)	152.0 ± 35.9	131.9 ± 6.5	156.3 ± 46.3	103.8 ± 7.1	163.0 ± 37.5	129.4 ± 22.5	157.7 ± 38.9	102.0 ± 42.6
Sugar (g)	24.9 ± 17.1	18.8 ± 1.1	25.6 ± 11.9	18.7 ± 3.8	23.4 ± 8.1	17.0 ± 10.0	22.2 ± 9.0	13.1 ± 6.3
Protein (g)	67.1 ± 14.4	48.4 ± 3.2	74.8 ± 10.5	37.8 ± 7.2	78.5 ± 14.0	56.1 ± 12.8	67.5 ± 10.8	53.7 ± 12.3
Fat (g)	50.0 ± 12.1	33.5 ± 6.3	49.6 ± 15.6	25.6 ± 3.3	57.5 ± 12.5	46.1 ± 11.6	47.1 ± 13.0	41.8 ± 14.5
Saturated fat (g)	12.1 ± 3.4	6.9 ± 2.1	11.2 ± 2.6	5 ± 0.7	14.8 ± 3.6	12.7 ± 4	11.8 ± 2.6	10.2 ± 5.8
Percent from Energy								
Carbohydrate (%)	47.0 ± 7.3	52.1 ± 3.1	45.6 ± 4.8	52.6 ± 3	44.3 ± 5.2	47 ± 6.1	48.2 ± 4.2	42.1 ± 14.1
Sugar (%)	6.7 ± 3.1	7.5 ± 0.4	7.6 ± 2.4	9.3 ± 1.5	6.1 ± 1.6	6.1 ± 3.6	6.8 ± 2.1	5.1 ± 1.8
Protein (%)	20.3 ± 2.8	18.7 ± 0.6	22.8 ± 6.0	18.7 ± 2.2	21.8 ± 2.1	19.3 ± 2.1	21.1 ± 2.8	22.1 ± 5.5
Fat (%)	32.7 ± 5.3	29.2 ± 3.5	31.6 ± 2.3	28.7 ± 2.2	33.9 ± 3.6	33.7 ± 4.2	30.7 ± 3.2	35.7 ± 9.3
Saturated fat (%)	7.7 ± 1.5	5.9 ± 1.2	7.3 ± 0.7	5.6 ± 0.5	8.6 ± 1.0	8.9 ± 1.4	7.7 ± 0.5	8.5 ± 2.7

## References

[1] Kim Y., Nho S.J., Woo G., Kim H., Park S., Kim Y., Park O., Oh K. (2021). Trends in the prevalence and management of major metabolic risk factors for chronic disease over 20 years: Findings from the 1998–2018 Korea National Health and Nutrition Examination Survey. Epidemiol. Health.

[2] Park S.E., Ko S.H., Kim J.Y., Kim K., Moon J.H., Kim N.H., Han K.D., Choi S.H., Cha B.S. (2025). Diabetes fact sheet in Korea 2024. Diabetes Metab. J..

[3] Yang Y.S., Han B.-D., Han K., Jung J.-H., Son J.W. (2022). Obesity fact sheet in Korea, 2021: Trends in obesity prevalence and obesity-related comorbidity incidence stratified by age from 2009 to 2019. J. Obes. Metab. Syndr..

[4] Hu F.B. (2011). Globalization of Diabetes. Diabetes Care.

[5] Ha K.H., Kim D.J. (2015). Trends in the Diabetes Epidemic in Korea. Endocrinol. Metab..

[6] Ley S.H., Hamdy O., Mohan V., Hu F.B. (2014). Prevention and management of type 2 diabetes: Dietary components and nutritional strategies. Lancet.

[7] Moon J.S., Kang S., Choi J.H., Lee K.A., Moon J.H., Chon S., Kim D., Kim H.J., Seo J.A., Kim M.K. (2024). 2023 clinical practice guidelines for diabetes management in Korea: Full version recommendation of the Korean diabetes association. Diabetes Metab. J..

[8] Goldenberg J.Z., Day A., Brinkworth G.D., Sato J., Yamada S., Jönsson T., Beardsley J., A Johnson J., Thabane L., Johnston B.C. (2021). Efficacy and safety of low and very low carbohydrate diets for type 2 diabetes remission: Systematic review and meta-analysis of published and unpublished randomized trial data. bmj.

[9] Malandrucco I., Pasqualetti P., Giordani I., Manfellotto D., De Marco F., Alegiani F., Sidoti A.M., Picconi F., Di Flaviani A., Frajese G. (2012). Very-low-calorie diet: A quick therapeutic tool to improve β cell function in morbidly obese patients with type 2 diabetes. Am. J. Clin. Nutr..

[10] Sathananthan M., Shah M., Edens K.L., Grothe K.B., Piccinini F., Farrugia L.P., Micheletto F., Man C.D., Cobelli C., A Rizza R. (2015). Six and 12 weeks of caloric restriction increases β cell function and lowers fasting and postprandial glucose concentrations in people with type 2 diabetes. J. Nutr..

[11] Zubrzycki A., Cierpka-Kmiec K., Kmiec Z., Wronska A. (2018). The role of low-calorie diets and intermittent fasting in the treatment of obesity and type-2 diabetes. J. Physiol. Pharmacol..

[12] Lim E.L., Hollingsworth K.G., Aribisala B.S., Chen M.J., Mathers J.C., Taylor R. (2011). Reversal of type 2 diabetes: Normalisation of beta cell function in association with decreased pancreas and liver triacylglycerol. Diabetologia.

[13] Steven S., Taylor R. (2015). Restoring normoglycaemia by use of a very low calorie diet in long-and short-duration Type 2 diabetes. Diabet. Med..

[14] Goday A., Bellido D., Sajoux I., Crujeiras A.B., Burguera B., García-Luna P.P., Oleaga A., Moreno B., Casanueva F.F. (2016). Short-term safety, tolerability and efficacy of a very low-calorie-ketogenic diet interventional weight loss program versus hypocaloric diet in patients with type 2 diabetes mellitus. Nutr. Diabetes.

[15] Kashyap A., Mackay A., Carter B., Fyfe C.L., Johnstone A.M., Myint P.K. (2022). Investigating the Effectiveness of Very Low-Calorie Diets and Low-Fat Vegan Diets on Weight and Glycemic Markers in Type 2 Diabetes Mellitus: A Systematic Review and Meta-Analysis. Nutrients.

[16] Lean M.E., Leslie W.S., Barnes A.C., Brosnahan N., Thom G., McCombie L., Peters C., Zhyzhneuskaya S., Al-Mrabeh A., Hollingsworth K.G. (2018). Primary care-led weight management for remission of type 2 diabetes (DiRECT): An open-label, cluster-randomised trial. Lancet.

[17] Taheri S., Zaghloul H., Chagoury O., Elhadad S., Ahmed S.H., El Khatib N., Amona R.A., El Nahas K., Suleiman N., Alnaama A. (2020). Effect of intensive lifestyle intervention on bodyweight and glycaemia in early type 2 diabetes (DIADEM-I): An open-label, parallel-group, randomised controlled trial. Lancet Diabetes Endocrinol..

[18] Muscogiuri G., El Ghoch M., Colao A., Hassapidou M., Yumuk V., Busetto L. (2021). European Guidelines for Obesity Management in Adults with a Very Low-Calorie Ketogenic Diet: A Systematic Review and Meta-Analysis. Obes. Facts.

[19] Song Y., Joung H. (2012). A traditional Korean dietary pattern and metabolic syndrome abnormalities. Nutr. Metab. Cardiovasc. Dis..

[20] Kim M.K., Kim J., Park S., Song Y., Kwon H. (2025). Impact of caloric restriction on diabetes remission in Korean adults with obesity (CREDO-K study). Diabetes Obes. Metab..

[21] Jensen M.D., Ryan D.H., Apovian C.M., Ard J.D., Comuzzie A.G., Donato K.A., Hu F.B., Hubbard V.S., Jakicic J.M., Kushner R.F. (2014). 2013 AHA/ACC/TOS Guideline for the Management of Overweight and Obesity in Adults. Circulation.

[22] Atkinson R.L., Dietz W.H., Foreyt J.P., Goodwin N.J., Hill J.O., Hirsch J., Pi-Sunyer X., Weinsier R.L., Wing R., Yanovsko S.Z. (1993). Very low-calorie diets. JAMA.

[23] Heilbronn L.K. (2006). Effect of 6-month calorie restriction on biomarkers of longevity, metabolic adaptation, and oxidative stress in overweight individuals: A randomized controlled trial. JAMA.

[24] Flanagan E.W., Spann R., Berry S.E., Berthoud H., Broyles S., Foster G.D., Krakoff J., Loos R.J.F., Lowe M.R., Ostendorf D.M. (2023). New insights in the mechanisms of weight-loss maintenance: Summary from a Pennington symposium. Obesity.

[25] Lean M.E.J., Leslie W.S., Barnes A.C., Brosnahan N., Thom G., McCombie L., Peters C., Zhyzhneuskaya S., Al-Mrabeh A., Hollingsworth K.G. (2019). Durability of a primary care-led weight-management intervention for remission of type 2 diabetes: 2-year results of the DiRECT open-label, cluster-randomised trial. Lancet Diabetes Endocrinol..

[26] Lean M.E.J., Leslie W.S., Barnes A.C., Brosnahan N., Thom G., McCombie L., Peters C., Zhyzhneuskaya S., Al-Mrabeh A., Hollingsworth K.G. (2024). A very-low-calorie diet (VLCD) intervention for the management of prediabetes and early Type 2 diabetes mellitus in a multi-ethnic cohort in Aotearoa New Zealand: The PROGRESS NZ feasibility study. Asia Pac. J. Clin. Nutr..

[27] Churuangsuk C., Hall J., Reynolds A., Griffin S.J., Combet E., Lean M.E.J. (2022). Diets for weight management in adults with type 2 diabetes: An umbrella review of published meta-analyses and systematic review of trials of diets for diabetes remission. Diabetologia.

[28] Choi J.H., Min S.H., Lim K.H., Shin U.J., Kim M.-S. (2020). Glucose-lowering effect of home-delivered therapeutic meals in patients with type 2 diabetes. J. Korean Diabetes.

[29] Aller E.E.J.G., Larsen T.M., Claus H., Lindroos A.K., Kafatos A., Pfeiffer A., Martinez J.A., Handjieva-Darlenska T., Kunesova M., Stender S. (2014). Weight loss maintenance in overweight subjects on ad libitum diets with high or low protein content and glycemic index: The DIOGENES trial 12-month results. Int. J. Obes..

[30] Kim J.Y. (2020). Optimal diet strategies for weight loss and weight loss maintenance. J. Obes. Metab. Syndr..

[31] Ha K., Joung H., Song Y. (2018). Low-carbohydrate diet and the risk of metabolic syndrome in Korean adults. Nutr. Metab. Cardiovasc. Dis..

[32] Goris A.H., Westerterp-Plantenga M.S., Westerterp K.R. (2000). Undereating and underrecording of habitual food intake in obese men: Selective underreporting of fat intake. Am. J. Clin. Nutr..

[33] Kanellakis S., Sidiropoulou S., Apostolidou E., Skoufas E., Bountouvi E., Prelorentzou T., Manios Y. (2021). Association of dietary intake underreporting with body image perception. Clin. Nutr. Open Sci..

